# Therapeutic Effects of Nanoparticles Loaded with Combined Sense and Anti-Sense of EpoR Plasmids to Enhance Angiogenesis

**DOI:** 10.21203/rs.3.rs-9718352/v1

**Published:** 2026-06-11

**Authors:** Linda Noukeu, Priyanka Iyer, Vy Tran, Amirhossein Hakamivala, Na Nguyen, Liping Tang, Kytai T. Nguyen, Tam Nguyen

**Affiliations:** Department of Bioengineering, The University of Texas at Arlington, Arlington, Texas 76010, USA

## Abstract

We employed nanoparticle (NP) platforms to enhance the delivery of both erythropoietin receptor (EpoR) plasmids (pEpoR) and the antisense of pEpoR (pRopE) to induce angiogenesis in ischemic tissues of peripheral arterial disease (PAD). Poly (lactic-co-glycolic acid) (PLGA) NPs loaded with these plasmids were successfully fabricated and characterized. In migration assays, pEpoR/pRopE NPs exhibited a synergistic effect, significantly outperforming pEpoR NPs alone (by 3.7-fold) and pRopE NPs alone (by 2.3-fold). Furthermore, the dual-plasmid NPs significantly enhanced cell survival and facilitated tube formation compared to the VEGF-treated group. In a PAD animal model, pEpoR/pRopE NPs improved blood perfusion in ischemic limbs by 3.8-fold and increased capillary densities in the gastrocnemius muscle by 4.7-fold compared to the VEGF group. In conclusion, pEpoR/pRopE NPs successfully transfected ischemic cells, enhancing both blood perfusion and capillary densities in ischemic tissues, highlighting their therapeutic potential for PAD treatment.

## Introduction

Peripheral arterial disease (PAD) is an impairment in arterial function, particularly within the femoral arteries, resulting in deficient blood and oxygen supplies to the lower extremities ^1^. PAD affect millions of individuals in the U.S. (~ 12 million), particulally elderly men over 60 years of age ^2,3^. PAD can progress to critical limb ischemia (CLI), which carries high risks of amputations (30% within 1 year) and mortalities (20–25% within 1 year and 40–50% within 5 years) ^4^. Recently, therapeutic angiogenesis has proven to be a promising strategy to mitigate patients’ symptoms, as it promotes blood vessel formation to supply oxygen-rich blood and nutrients to deficit tissues ^5^. Gene therapy is one of the most common approaches utilized to stimulate therapeutic angiogenesis ^6^. Among pro-angiogenic plasmids, vascular endothelial growth factor (VEGF), fibroblast growth factor (FGF), hepatocyte growth factor (HGF), stromal derived growth factor (SDF), and platelet derived growth factor (PDGF) has been most extensively studied in preclinical and clinical trials; however, they have failed to demonstrate benefits regarding primary outcomes in clinical trials ^7–9^. Therefore, a clear need remains to develop a novel strategy to enhance clinically effective angiogenesis for the treatment of PAD.

The pEpoR plasmid has been identified as a pro-angiogenic agent that acts via upregulating the expression of VEGF and VEGFR in non-hematopoietic cells, such as endothelial cells (ECs). ^10–14^ EpoR expression triggers the Epo/EpoR signaling pathway, which regulates vasodilators/vasoconstrictors, modulates vascular tone synthesis, elicits pro-angiogenic programs in stem cells, and initiates angiogenic signaling pathway.^15–17^ EpoR, a 66–78 kDa transmembrane protein upregulated in hypoxic ECs ^18,19^, possesses cytoprotective properties against apoptosis and mediates angiogenic effects of Epo. ^20^ Meanwhile, RopE has been reported to be concurrently upregulated with EpoR expression *in vivo* following pneumonectomy.^21^ Combined treatment with RopE and EpoR transcripts has been shown to generate a synergistic upregulation of EpoR expression *in vitro* and in a canine lung model. ^22^ Although pRopE plays important roles in modulating EpoR expression, little is known about whether co-delivery of pEpoR/pRopE enhances these synergistic effects on therapeutic angiogenesis.

The delivery of naked plasmids can be performed via direct injection or viral vectors.^23,24^ However, these techniques are limited by low therapeutic efficiency and extensive side effects. ^25^ Poly (lactic-co-glycolic) acid (PLGA) is an FDA approved biodegradable polymer with good encapsulation properties that provides controlled, sustained release of its payloads ^26^, while polyethylenimine (PEI) is a polymer with outstanding transfection properties due to its ability to form complexes with plasmids through electrostatic interactions, resulting in higher transfection efficiency. In this paper, we formulated pEpoR/pRopE NPs to evaluate angiogenesis in *in vitro* studies and blood perfusion *in vivo* mouse PAD models. The novelty of this work was established by delivering a complementary strand of the same gene loaded onto PLGA NPs to investigate its synergistic therapeutic effects.

## Results

### Characterization of nanoparticles

**Figure 1:**
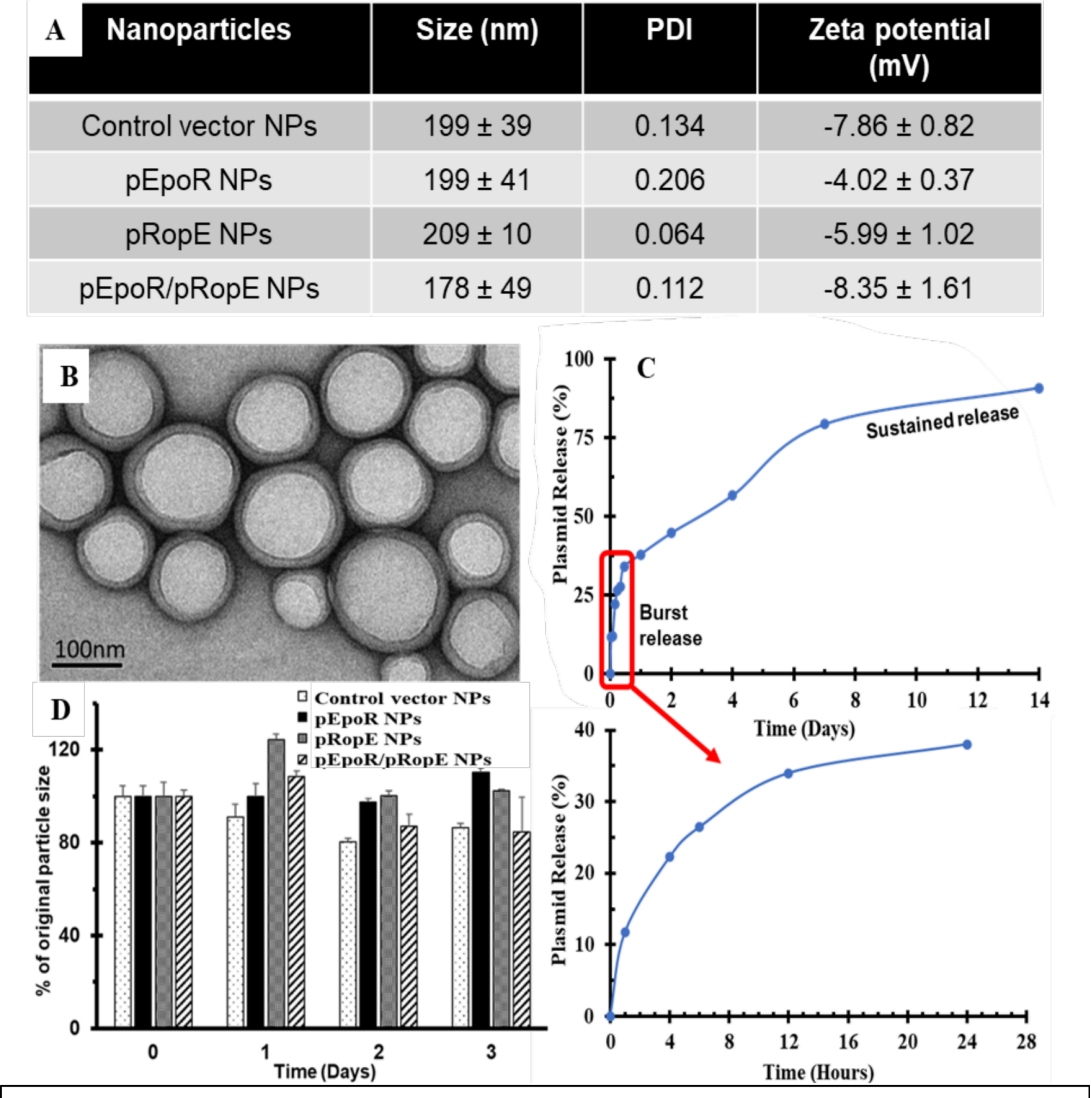
Nanoparticle characterization. A) DLS table of various NPs. B) Representative TEM images of pEpoR/pRopE NPs. C) Plasmid release profile of pEpoR/pRopE NPs: The top graph shows a biphasic release of NPs, whereas the bottom graph shows a zoomed-in view of the initial burst release phase. D) NP stability in saline (0.09%) showed that the NPs maintained their original size over a course of 3 days.

Dynamic light scattering (DLS) results showed that the average diameters of control vector NPs, pEpoR NPs, pRopE NPs, and pEpoR/pRopE NPs were 199 nm (PDI = 0.134), 199 nm (PDI = 0.206), 209 nm (PDI = 0.064), and 178 nm (PDI = 0.112), respectively (Fig. 1A). Zeta potential ranged from − 4.02 mV to −8.35 mV depending on the type of NPs. The plasmid loading efficiency was approximately 80%. Figure 1B shows representative TEM images of pEpoR/pRopE NPs, displaying spherical shapes with diameters ranging from 100nm to 150 nm. The plasmid release profile showed a biphasic curve with a 35% burst release in the first 12 hours, followed by a sustained release over 14 days (Fig. 1C). The stability study demonstrated that these NPs maintained their original sizes in saline for up to 72 hours (Fig. 1D).

### Cellular Uptake and Transfection Study of Nanoparticles

**Figure 2:**
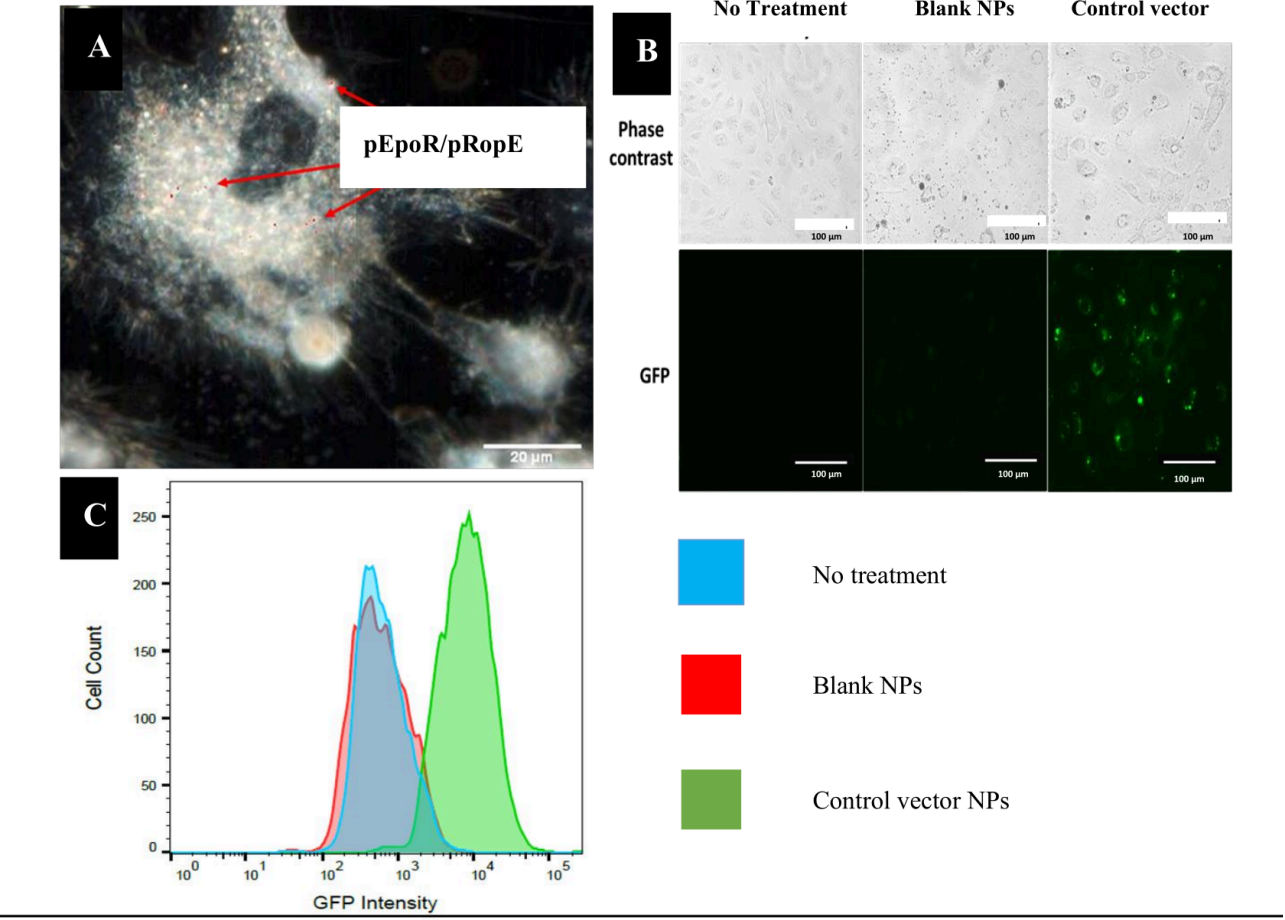
Cellular uptake and transfection study of NPs on HUVECs. **A)** Cytoviva images showing pEpoR/pRopE NPs endocytosed by HUVECs**. B)** Transfection efficiency of NPs in HUVECs on day 3. The results demonstrated that control vector NPs successfully transfect HUVECs and expressed GFP in the cytosol. The blank NPs served as negative control. Scale bar = 100 μm**. C)** Flow cytometry analysis showing a shift change of GFP intensities for control vector NPs group compared to the untreated and blank NPs groups.

Cytoviva hyperspectral imaging results showed that pEpoR/pRopE NPs were endocytosed into the HUVEC cytosol after 4 hours of incubation (Fig. 2A). Green fluorescent protein (GFP) expression was observed in HUVECs transfected with control vector NPs using a fluorescence microscope (Fig. 2B) and flow cytometry (Fig. 2C). Flow cytometry analysis showed a distinct shift in the GFP peak for the control vector NPs group compared to the blank NPs group (which showed no change). The results confirmed that HUVECs were successfully transfected and expressed the plasmid-encoded genes delivered via the NPs platform.

### In Vitro Therapeutic Efficacy of NPs in HUVECs

**Figure 3:**
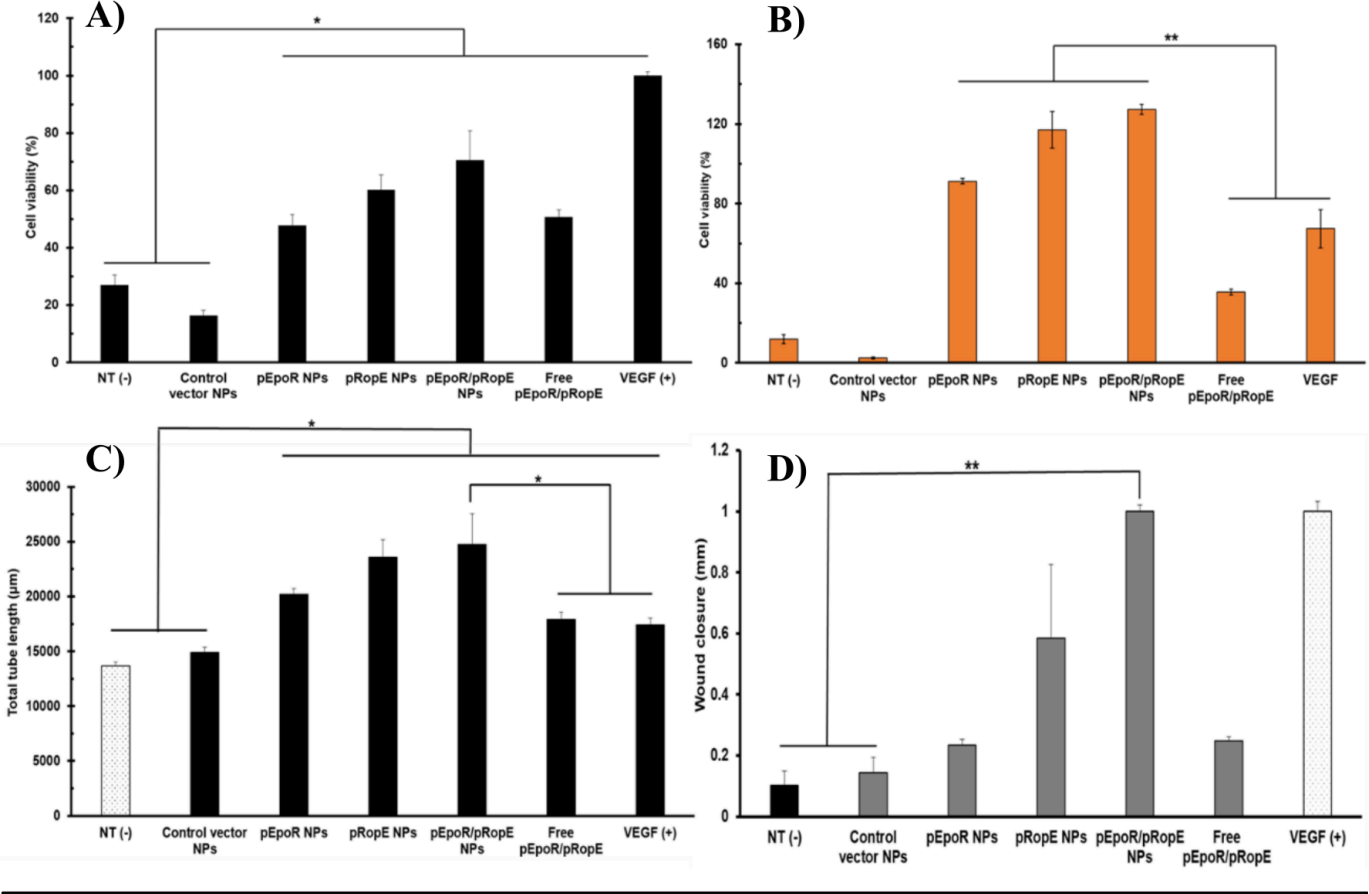
*In vitro* therapeutic efficacy of NPs in HUVECs. **A)** The proliferation study under hypoxic condition showed that cells treated with pEpoR/pRopE NPs, pEpoR NPs, pRopE NPs, free pEpoR/pRopE and VEGF significantly supported HUVEC proliferation compared to the no treatment (NT) and control vector NPs groups. **B)** The cyto-protection assay under an H_2_O_2_ environment demonstrated that pEpoR NPs, pRopE NPs, and pEpoR/pRopE NPs significantly protected HUVECs under oxidative stress compared to the VEGF and free pEpoR/pRopE groups. Cell viability was normalized to 100% using the VEGF group for Experiment A and B. **C)** The quantitative analysis data of tube formation length showed pEpoR/pRopE NPs significantly induced 1.8-fold and 1.7-fold increase in tube length compared to the no treatment and control vector NPs groups, respectively. Tube formation in the pEpoR/pRopE NPs group was also significantly increased compared to the free pEpoR/pRopE and VEGF groups. **D)** In the scratch assay, HUVECs pre-transfected with pEpoR/pRopE NPs migrated significantly faster than those treated with pEpoR NPs (by 3.8-fold) or pRopE NPs (by 2.3-fold). All data are represented as mean ± SEM (n=4). Cells without treatment and those treated with VEGF served as negative and positive controls, respectively. * (P≤0.05) and ** (P≤0.01) symbols indicate significant differences.

Pre-transfected HUVECs with pEpoR/pRopE NPs proliferated significantly more, showing a 2.7-fold and 4.7-fold increase compared to the NT and control vector NPs groups, respectively (Fig. 3A). Furthermore, cells treated with pEpoR/pRopE NPs exhibited faster growth rates than the free pEpoR/pRopE, pEpoR NPs, and pRopE NPs groups. Pre-transfection of HUVECs with pEpoR/pRopE NPs also significantly enhanced cell survival under oxidative stress compared to all other treatments (Fig. 3B). Specifically, pEpoR/pRopE NPs improved the viability of damaged cells by 11-fold versus NT, 4-fold versus free pEpoR/pRopE plasmids, 2-fold versus VEGF, and 1.4-fold versus pEpoR NPs. The tube formation assay demonstrated that the pEpoR/pRopE NPs sprouted the highest total tube length among all experimental groups (Fig. 3C). The pEpoR/pRopE NPs induced significantly higher angiogenic sprouting compared to both VEGF and free pEpoR/pRopE plasmids. Specifically, pEpoR/pRopE NPs promoted angiogenesis 2-fold more compared to the negative control groups and 1.4-fold more compared to the free pEpoR/pRopE and VEGF groups. Finally, in the scratch assay, HUVECs pre-transfected with pEpoR/pRopE NPs migrated faster to close the scratch wound area compared to the NT, control vector NPs, pEpoR NPs, pRopE NPs, and free pEpoR/pRopE group (Fig. 3D). Importantly, pEpoR/pRopE NPs demonstrated clear synergistic effects, migrating significantly faster than the pEpoR NPs (by 3.8-fold) and pRopE NPs (by 2.3-fold) monotherapy groups.

### In Vitro Cytotoxicity of NPs in HUVECs

**Figure 4:**
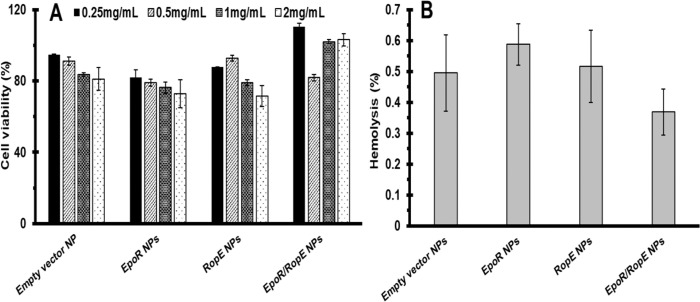
*In vitro* cytotoxicity study of NPs. **(A)** Cyto-compatibility assay and **(B)** hemo-compatibility assay of various plasmid-loaded NPs at different concentrations. The results showed that these NPs had no adverse effects on cell viability and caused less than 5% hemolysis when exposed to human blood.

The cytotoxicity of synthesized nanoparticles was assessed in HUVECs over 24 hours at different concentrations to determine the potential toxicity of these plasmids-loaded NPs (Fig. 4). The results showed that HUVECs maintained greater than 80% viability when exposed to 0.25 to 2.0 mg/ml NPs suspensions for 24 hours (Fig. 4A). When exposed to whole blood, less than 1% red blood cell lysis was observed (Fig. 4B). These results confirm that control vector NPs (empty vector NPs), pEpoR NPs, pRopE NPs, and pEpoR/pRopE NPs are all hemo-compatible and safe for subsequent evaluation in animal studies.

### In Vivo Therapeutic Effects of pEpoR/pRopE NPs on PAD Mice

**Figure 5:**
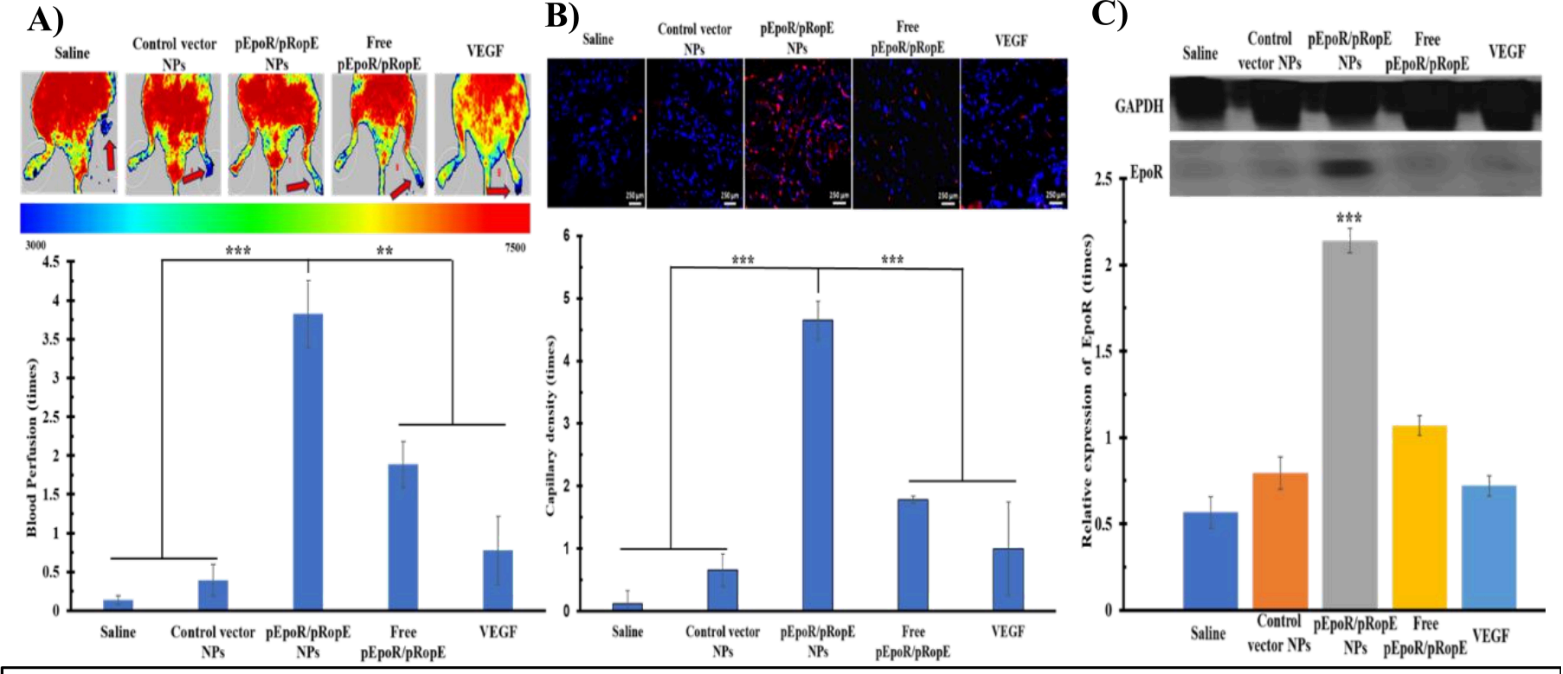
*In vivo* therapeutic efficacy study. **A)** pEpoR/pRopE NPs promoted blood perfusion on PAD models. The top panel shows representative images of blood perfusion on day 14^th^ post-treatment (red arrows), while the bottom panel shows their quantitative data. Compared to blood perfusion measured pre-treatment (day 0) within the same group, the results showed that pEpoR/pRopE NPs significantly restored blood perfusion by 3.8-fold, whereas free pEpoR/pRopE plasmids and VEGF only improved perfusion by 1.8-fold and 1.20-fold, respectively. **B)** pEpoR/pRopE NPs increased capillary densities in the ischemic gastrocnemius muscle. The top panel shows representative images of double immunofluorescent staining for CD31 (red) and DAPI (blue). The pEpoR/pRopE NPs group showed the significantly highest number of CD31+ cells compared to the other groups. The bottom panel shows the quantitative analyzed data of capillary density in the ischemic muscles. The results showed that pEpoR/pRopE NPs significantly improved capillaries density by 4.7-fold compared to VEGF treatment. No improvement was observed in the saline and control vector NPs groups. **C)** EpoR expression in ischemic gastrocnemius muscle. The panel shows representative cropped western blot images of GAPDH and EpoR protein, and the bottom shows the densitometric quantification of EpoR expression in the muscles. The original full blot images were presented in supplemental information. The results showed EpoR expression in pEpoR/pRopE NPs group increased 2.2-fold compared to free pEpoR/pRopE plasmids. All data are represented as mean ± SEM (n=3–5). * (P≤0.05), ** (P≤0.01), and *** (P≤0.001) symbols indicate significant differences.

Laser speckle contrast imaging (LSCI) was utilized to monitor the improvement of blood perfusion post-treatment (Fig. 5A). Compared to blood perfusion measured on day 0, PAD mice treated with pEpoR/pRopE NPs demonstrated a significantly 3.8-fold increase in blood perfusion. Free pEpoR/pRopE plasmids and VEGF enhanced blood perfusion in the ischemic limbs by only 1.8-fold and 1.2-fold, respectively. Statistical analysis demonstrated that pEpoR/pRopE NPs significantly enhanced blood perfusion compared to both free pEpoR/pRopE plasmids and VEGF. In contrast, blood perfusion progressively declined over the course of treatment in the saline and control vector NPs groups.

To confirm the efficacy of pEpoR/pRopE NPs in stimulating angiogenesis, we assessed EC signaling, adhesion, and motility during vascular reconstruction by staining for CD31^+^ expression in ischemic gastrocnemius muscles (Fig. 5B). As expected, pEpoR/pRopE NPs treatment resulted in a significant increase in CD31^+^ capillary structures (red) within the hypoxic muscles compared to all other groups. Specifically, capillary densities in the pEpoR/pRopE NPs group increased 4.7-fold versus VEGF treatment and 2.6-fold versus free pEpoR/pRopE plasmids, whereas minimal capillary densities were observed in the negative control groups, including the saline and control vector NPs. Western blot analysis showed that EpoR expression in dual-NP groups significantly increased by more than 2-fold compared to the free pEpoR/pRopE plasmid group. No significant differences in expression were detected among the VEGF, control vector NPs, and saline groups.

## Discussion

Gene therapy is being investigated as a potential approach for tissue regeneration and blood flow reperfusion for PAD treatment; however, its major limitations include poor cell membrane penetration, higher enzymatic degradation, and immunogenicity concerns, which ultimately contribute to high amputation rates in CLI patients.^27,28^ To overcome these challenges, we applied NPs as carriers for the dual delivery of pEpoR/pRopE to treat PADs. To enhance the transfection efficiency of the plasmids, we initially complexed them with PEI polymers via electrostatic bonding. It should be noted that PEI transfection efficiency and cytotoxicity are heavily dependent on its molecular weight ^29,30^. In this study, we used branched, low-molecular-weight (1.2kDa) PEI to induce a “proton sponge” effect, thereby facilitating escape from lysosomal degradation and improving plasmid translocation to the nucleus.^31^ PLGA, an FDA-approved, biodegradable polymer, possesses good encapsulation and degradation properties. ^26^ The release rate profile of pEpoR/pRopE NPs supports the minimum duration required to stimulate functional angiogenesis ^32^; although the specific ratio and amount of each plasmid released *in vivo* remains unclear and should be determined in future investigations. Furthermore, our cytotoxicity studies showed minimal adverse effects, confirming that these platforms are safe for subsequent pre-clinical and clinical studies.

We achieved significantly higher levels of cytoprotection using pEpoR NPs, pRopE NPs, and pEpoR/pRopE NPs. The EC protection afforded by these NPs in a stressed environment may involve several distinct mechanisms, including the inactivation of pro-inflammatory cytokines such as tumor necrosis factor (TNF) ^33^ and the downregulation of inflammatory cascades like the p38 MAPK pathway.^34^ The nitric oxide (NO) signaling pathway might also be critically involved in the cytoprotective effects of pEpoR and pRopE under stress conditions. Although the complex nature of angiogenesis has yet to be fully revealed, cell migration remains an essential component of the process. The EpoR/Epo pathway is known to be involved in the mobilization of endogenous progenitor cells during active angiogenesis.^35^ It is also well documented that a regulatory link exists between the EpoR/Epo pathway and upstream VEGF expression.^36^ In the current work, we observed a synergistic boost in EC migration driven by pEpoR/pRopE NPs, which demonstrated outstanding performance even when compared to the VEGF-treated group. This enhancement in migration velocity by pEpoR/pRopE NPs was significantly higher than that observed in the NT or control vector NPs groups.

When ECs are exposed to hypoxia, the upregulation of various angiogenic proteins is prominent, driving an increase in endothelial tube formation^37^ and rendering neo-vessel assembly inevitable. For instance, 28 days post-administration of VEGF plasmid-loaded NPs in mice, a higher microvascular density was observed compared to groups treated with saline or naked VEGF plasmids.^38^ In addition, VEGF is a context-dependent growth factor that often requires the cooperative input of other proteins or nucleic acids to achieve mature, sustainable angiogenesis.^39^ EpoR expression is known to directly influence endogenous VEGF levels. ^40^ The robust increase in tube formation observed with our pEpoR and pRopE formulation can be explained by the augmented bioavailability of pEpoR and pRopE within the targeted cells, triggering a strong positive feedback mechanism in the EpoR pathway that results in enhanced local VEGF production, as observed in our study and others.^21^ Recently, co-delivery of a VEGF plasmid/apelin NPs resulted in higher and more consolidated tube formation than that of the VEGF plasmid alone ^41^, whereas the dual-delivery of bFGF and VEGF nanogels to endothelial progenitor cells (EPC) did not yield a corresponding increase in tube formation.^42^ This confirms that despite being primary drivers of angiogenesis, absolute VEGF and FGF efficiency depends heavily on complementary co-factors. Although VEGF is widely recognized for its mitogenic potential, ability to stimulate angiogenesis, and capacity to inhibit apoptosis ^43,44^, corresponding clinical trials have historically failed to demonstrate substantial benefits regarding primary outcomes in PAD patients.^45^

In summary, we have reported for the first time the application of an NP platform for the co-delivery of pEpoR/pRopE to treat PADs. We demonstrated that 1) PLGA NPs are stable, hemocompatible, cytocompatible, and serve as an effective transfection platform with clear biphasic sustained-release properties; and 2) pEpoR/pRopE NPs significantly improve blood perfusion by enhancing angiogenesis both *in vitro* and in vivo in a model model of PAD. Our future study will investigate the explicit downstream molecular mechanisms and pharmacokinetics of pEpoR/pRopE NPs in PAD mice to further map the therapeutic angiogenesis pathway involved.

## Methods

### Cell Culture

Human umbilical vein endothelial cells (HUVECs) were purchased from ATCC and cultured in Vasculife^®^ VEGF (LS-1020) complete media (Lifeline^®^ Cell Technology) with 1% Penicillin and Streptomycin (Life Technologies) at 37°C and a 5% CO_2_ atmosphere. Conditioned media (CM) containing 2% fetal bovine serum (FBS) (GElifescience) was used for HUVECs across all *in vitro* studies and assays.

### Nanoparticle Fabrication

Different plasmids were purified using EndoFree Plasmid Giga Kits (cat. no. 12391) following the manufacturer’s (Qiagen) instructions.^46^ PEI-plasmid complexes were prepared via an electrostatic complexation method.^47^ Briefly, 0.15 mg of branched PEI (bPEI, 1,2kD) at 0.05% (w/v) was added to the plasmids/glucose (5 mg/5 mg) solution and allowed to complex at room temperature for 30 minutes under rotation. A standard double emulsion solvent evaporation technique was applied to fabricate NPs.^48–51^ Plasmid-loaded NPs were collected using centrifugation at 20,000 rpm for 15 minutes. The resulting pellets were vigorously resuspended in 2 mL deionized (DI) water, lyophilized to form the powder products, and stored at −20°C for subsequent experiments. The fabrication protocol was performed under sterilized conditions; thus, the NPs suspension was safe to use both *in vitro* and *in vivo* experiments.

### Nanoparticle Characterization

Transmission electron microscopy (TEM) was used to confirm the NP morphology. A NanoBrook 90 Plus Zetal PAL instrument (Brookhaven Instruments Co.) was utilized for particle size and zeta potential measurements. NP stability in saline (0.9%) was monitored for 48 hours, as previously described.^52,53^ Release profiles were determined using a centrifugal method of NP suspensions (1 mg/ml in PBS) placed in a shaking incubator at 150 RPM at 37°C at specific timepoints (0h, 1h, 3h, 6h, 12h, 24h, 2d, 5d, 7d, and 14d). Ultraviolet (UV) absorbance at 260/280 nm via spectrophotometry (Tecan) was used to quantify released plasmids. Lastly, un-entrapped plasmids collected from the supernatant during NPs fabrication were quantified to determine plasmid loading efficiency, calculated as the percentage of the initial amount used (5 mg plasmids) according to Eq. 1.


(1) $$Loading\;efficiency\;=\frac{\mathrm{plasmids}\;\mathrm{used}-\mathrm{plasmids}\;\mathrm{in}\;\mathrm{supernatant}}{\mathrm{plasmids}\;\mathrm{used}}100\%$$

### Cell transfection using plasmid-loaded NPs

HUVECs were seeded at 500,000 cells/well on 6 well-plates overnight. Cells were starved in VascuLife^®^ Basal media for 2 hours before being incubated with plasmid-loaded NPs in Opti-MEM solutions for 2 hours. These cells were then washed and replenished with complete medium to determine transfection efficiency, using green fluorescent protein (GFP) expression as a transfected reported marker. Images of the transfected cells were captured over time using a fluorescent microscope.

### Proliferation study

Pre-transfected HUVECs were seeded at 4,000 cells/well on 96-well plates before incubation with conditioned media (CM) containing various therapeutic formulations under hypoxic conditions (< 1% O_2,_ 5% CO_2_ in nitrogen balance at 37°C) for 3 days. Cells treated with CM containing 25 ng/mL VEGF served as positive controls, while cells cultured in CM served as negative controls. MTS assays were performed to analyze the number of viable cells following the manufacturer’s (Promega) instructions.

### Cytoprotection assay

The cell protection experiment was carried out as described earlier ^54^. Briefly, pre-transfected HUVECs were seeded at 15,000 cells/well in 96-well plates, washed, and replenished with 200 μmol/L H_2_O_2_ in CM for 1 day. Cells exposed to H_2_O_2_ without therapeutic treatment served as the negative control, and those treated with 25 ng/mL VEGF served as the positive control. Cell viability was evaluated via the MTS assay.

### Tube formation assay

Tubular structure formation on Matrigels was performed as previously described. ^55–57^ Pre-transfected HUVECs were seeded at 15,000 cells/well onto growth factor-reduced basement membrane Matrigels matrices with basal media in 48-well plates. Cells were incubated under hypoxic conditions (< 1% O_2,_ 5% CO_2_ in nitrogen at 37°C) during the experiment. Non-transfected cells and cells exposed to 25 ng/mL of VEGF served as negative and positive controls, respectively. Phase-contrast images were captured to evaluate tube formation at 12 hours. Quantitative analysis of the total capillary sprouting length and density was performed using National Institutes of Health (NIH) ImageJ analysis software.

### Migration assay

EC migration capability was quantified using a published scratch wound procedure.^58^ Briefly, pre-transfected HUVECs were seeded at 15,000 cells/well on 96-well plates overnight before a scratch wound was created using a 1000 uL pipette tip. The cells were washed with 1x PBS to remove debris and incubated in basal media with different treatments for 24 hours at 37°C. The initial distance at 0 hours and the wound closure distance at 24 hours were documented using phase-contrast microscopy images. Cells without treatment in CM served as a negative control, while cells exposed to media containing 25 ng/mL VEGF were used as the positive control. Cell migration was quantified as the migrated distance relative to the initial distance.

### Cytotoxicity Studies

The hemocompatibility of NPs was assessed using an established procedure ^59^. Briefly, NPs suspensions at various concentrations (0 to 1 mg/mL) were incubated with human blood. Blood clotting kinetics were recorded over time (0 to 60 minutes) by measuring optical absorbance at 540 nm using a UV-Vis spectrophotometer, as previously described ^59^. Furthermore, a hemolysis test was performed by exposing saline-diluted blood (0.9% w/v; Sigma-Aldrich) to the NPs and measuring the absorbance of the supernatant at 545 nm using a UV-Vis spectrophotometer ^59^. The absorbance was converted and calculated to determine the percentage of hemolysis using Eq. 2.


(2) $$\%\;hemolysis=\frac{\left[(\mathrm{absorbance}\;\mathrm{of}\;\mathrm{tested}\;\mathrm{NPs})-(\mathrm{absorbance}\;\mathrm{of}\;(-)\;\mathrm{control})\right]}{\;\mathrm{absorbance}\;\mathrm{of}\;(+)\;\mathrm{control}}*100$$

The cytocompatibility of the NPs was evaluated using a cell culture system as described earlier.^59^ HUVECs were seeded in culture plates and treated with various concentrations (0 to 2 mg/mL) of NPs for 24 hours. Cell viability and cytotoxicity were assessed via MTS cell proliferation and lactate dehydrogenase (LDH) leakage assays, respectively.

### In Vivo Evaluation of NPs in PAD Mice

All animal procedures were approved by the Institutional Animal Care and Use Committee (IACUC) at the University of Texas at Arlington (UTA). Balb/c mice were purchased from Charles River Laboratories (Wilmington, MA) and housed at the UTA animal facility under standard conditions with a regular chow diet until they reached 6 to 8 months of age for experimentation. An established PAD model was generated as previously described ^60^. Briefly, mice with their left thighs shaved 24 hours prior to surgery were intramuscularly injected with buprenorphine (1μg buprenorphine/g animal weight) for analgesia. Anesthesia was induced in an isoflurane induction chamber and maintained with 1% to 3% isoflurane in oxygen at a flow rate of 1 to 2 L/min using a Moduflex Compact Veterinary Anesthesia Machine (Dispomed Ltd., Canada). The mice were securely positioned supine, and the surgical site on the left thigh was prepared by cleaning three times with alternating iodine and 70% alcohol solution. To create the hindlimb ischemia model, a 1 cm skin incision was made in the upper anterior thigh region, and the femoral artery along with its major branches (proximal caudal femoral artery and superficial caudal epigastric artery) was ligated. The skin incisions were closed using 7 – 0 absorbable sutures. All animals were allowed to fully recover on heating pads before being returned to their assigned cages.

Following a 5-day post-operative recovery period, the PAD mice were randomly assigned to therapeutic study groups. Mice were given an intramuscular injection into the ischemic gastrocnemius muscles containing a 50 μL volume of their respective treatment using a 31 G needle. The experimental groups consisted of: saline, control vector NPs (2 mg/mouse), pEpoR/pRopE NPs (2 mg/mouse), free pEpoR/pRopE plasmids, and VEGF (3 μg/mouse). Dynamic blood perfusion was monitored non-invasively using laser speckle contrast imaging (LSCI) (PeriFlux 6000 Laser Doppler, Perimed).^61^ The blood perfusion ratio of the ischemic limb (left) to the non-ischemic limb (right) was calculated to quantify functional recovery.

At the experimental endpoints, mice were euthanized via CO_2_ exposure followed by cervical dislocation to ensure death, in strict accordance with the recommendations of the Panel on Euthanasia of the American Veterinary Medical Association and approved by our IACUCs. The gastrocnemius muscles of both healthy and ischemic legs were harvested and processed for histological staining. Tissue cross-sections (10 μm thick) were cut and stained for the endothelial marker CD31 to evaluate capillary density.^62^ EpoR expression in ischemic muscles was analyzed using western blot assay as described.^63^ Briefly, protein lysates were separated by SDS-PAGE gel electrophoresis and transferred onto nitrocellulose membrane (Biorad). The membranes were blocked and incubated with primary and secondary antibodies against EpoR and Glyceraldehyde-3-Phosphate Dehydrogenase (GAPDH) as a loading control. Target band signal intensities were quantified using NIH ImageJ software.

### Data Analysis

The research data was compiled in Microsoft Excell. Statistical analysis data are presented as means ± S.E.M. Two-way ANOVA tests were performed to compare differences among multiple groups, and a value of *P* < 0.05 was considered statistically significant.

## Supplementary Material

Supplementary Files

This is a list of supplementary files associated with this preprint. Click to download.

• Supplementalinformation.pdf
